# Template or ligand? Different structural behaviours of aromatic amines in combination with zinco­phosphite networks

**DOI:** 10.1107/S2056989018012343

**Published:** 2018-09-11

**Authors:** William Holmes, David B. Cordes, Alexandra M. Z. Slawin, William T. A. Harrison

**Affiliations:** a Department of Chemistry, University of Aberdeen, Meston Walk, Aberdeen AB24 3UE, Scotland; b School of Chemistry, University of St Andrews, Fife KY16 9ST, Scotland

**Keywords:** zinc phosphite, ligand, template, disorder, crystal structure

## Abstract

The solution-mediated syntheses and crystal structures of *catena*-poly[bis­(2-amino-3-hy­droxy­pyridinium) [zinc-di-μ-phospho­nato] dihydrate], {(C_5_H_7_N_2_O)[Zn(HPO_3_)_2_]·2H_2_O}_*n*_, (I), and poly[(benzene-1,2-di­amine)(μ_5_-phospho­nato)zinc], [Zn(HPO_3_)(C_6_H_8_N_2_)]_*n*_, (II) are described. The extended structure of (I) features [010] anionic chains of vertex-sharing ZnO_4_ tetra­hedra and HPO_3_ pseudopyramids; these chains are characterized by disorder over major [occupancy 0.7962 (13)] and minor [0.2038 (13)] components, which can be superimposed on each other by a nominal translational shift. The 2-amino-3-hy­droxy­pyridinium cations and water mol­ecules of crystallization inter­act with the ZnPO chains by way of numerous O—H⋯O and N—H⋯O hydrogen bonds. The structure of (II) features a direct Zn—N bond to the neutral 1,2-di­amino­benzene species as part of ZnO_3_N tetra­hedra as well as HPO_3_ pseudopyramids. The Zn- and P-centred groupings are linked through their O-atom vertices into infinite (010) sheets and the structure is consolidated by N—H⋯O hydrogen bonds and N—H⋯π inter­actions. The crystal of (I) chosen for data collection was found to be an inversion twin in a 0.56 (2):0.44 (2) domain ratio.

## Abstract 2

The solution-mediated syntheses and crystal structures of *catena*-poly[bis­(2-amino-3-hy­droxy­pyridinium) [zinc-di-μ-phospho­nato] dihydrate], (I), and poly[(benzene-1,2-di­amine)(μ_5_-phospho­nato)zinc], [Zn(HPO_3_)(C_6_H_8_N_2_)]_*n*_, (II) are described. The extended structure of (I) features [010] anionic chains of vertex-sharing ZnO_4_ tetra­hedra and HPO_3_ pseudopyramids while that of of (II) features a direct Zn—N bond to the neutral 1,2-di­amino­benzene species as part of ZnO_3_N tetra­hedra as well as HPO_3_ pseudopyramids.

## Chemical context   

Organically templated zinc phosphites (ZnPOs) are a well-established family of organic/inorganic open frameworks (*e.g*. Harrison *et al.*, 2001 ▸; Dong *et al.*, 2015 ▸; Huang *et al.*, 2017 ▸). The stated motivations for studying these phases include their potential applications in catalysis, separation and as ‘functional materials’ (Wang *et al.*, 2003 ▸). Important features of their crystal structures include the nature of the polyhedral building units [ZnO_4_, ZnO_3_(H_2_O), ZnO_3_N, HPO_3_] and their connectivity, which defines the Zn:P ratio; for example, ZnO_4_ and HPO_3_ units sharing all their vertices as Zn—O—P bonds will lead to an anionic [Zn_3_(HPO_3_)_4_]_*n*_^2*n*−^ framework (a 3:4 Zn:P ratio), the charge of which must be balanced by the organic templating cation (*e.g.* Katinaitė & Harrison, 2017 ▸). If, however, one of the P—O vertices is ‘terminal’ (a formal P=O double bond that does not link to zinc), then a [Zn(HPO_3_)_2_]_*n*_^2*n*−^ stoichiometry (1:2 Zn:P ratio) arises (*e.g.* Halime *et al.*, 2011 ▸). A combination of HPO_3_ (all vertices bonding) and HPO_3_ (one terminal vertex) units leads to a [Zn_2_(HPO_3_)_3_]_*n*_^2*n*−^ framework (2:3 Zn:P ratio) (Lin *et al.*, 2004*a* ▸). Another important structural feature of these phases is the ‘dual character’ of the organic species: most commonly it is a protonated organic amine, which inter­acts with the ZnPO framework *via* N—H⋯O hydrogen bonds (*e.g.* Harrison & McNamee, 2010 ▸). However, direct Zn—N bonds are also possible (*e.g*. Fan *et al.*, 2005 ▸), in which case the (unprotonated) organic species could be said to be acting as a ligand, although its steric bulk means that it does exert a ‘templating effect’ on the extended structure. This has an important effect on the zinc-to-phospho­rus ratio; for example, a combination of ZnO_3_N and HPO_3_ (all vertices bonding) units leads to a neutral [Zn(HPO_3_)]_*n*_ (1:1 Zn:P ratio) network (*e.g*. Lin *et al.*, 2004*b* ▸). The complex structure of {(C_4_H_12_N_2_)[Zn_5_(HPO_3_)_6_(C_4_H_10_N_2_)]}_*n*_ (Harrison, 2006 ▸) is notable for featuring the same organic species acting as a protonated template and a ligand in the same structure.
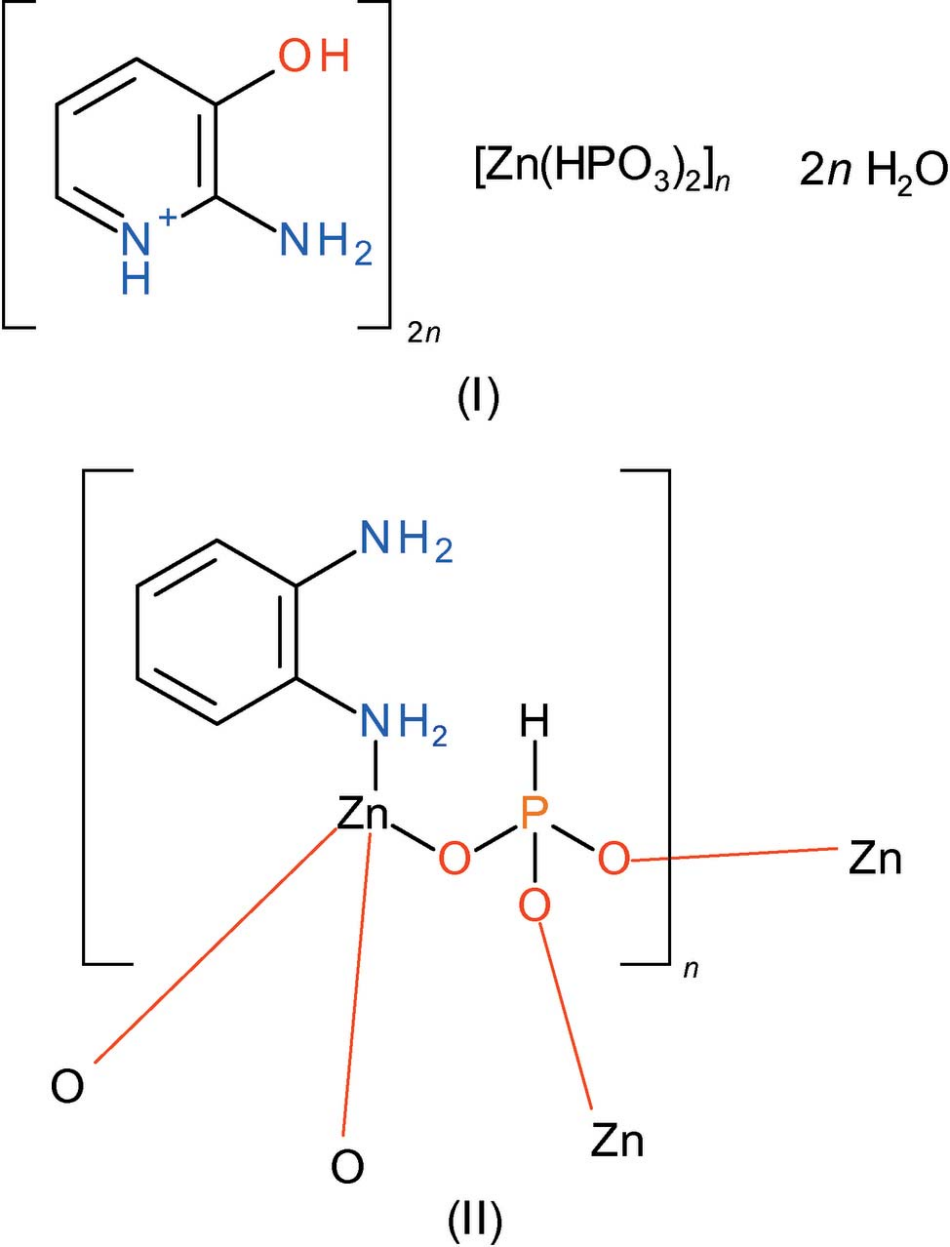


As part of our ongoing studies in this area we now describe the syntheses and structures of (C_5_H_7_N_2_O)[Zn(HPO_3_)_2_]·2H_2_O, (I), and [Zn(HPO_3_)(C_6_H_8_N_2_)], (II), where C_5_H_7_N_2_O^+^ is the 2-amino-3-hy­droxy­pyridinium cation and C_6_H_8_N_2_ is neutral 1,2-di­amino­benzene (also known as *o*-phenyl­enedi­amine).

## Structural commentary   

**Figure 1:**
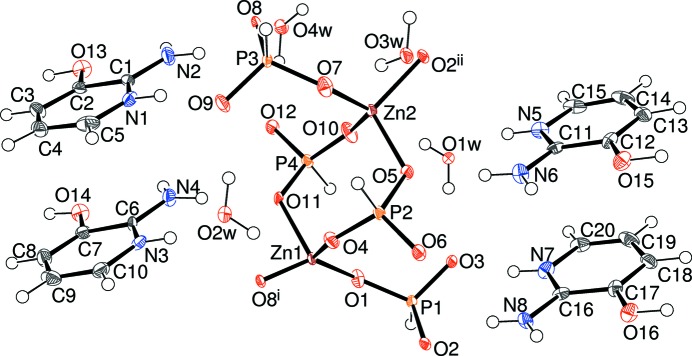
The asymmetric unit of (I) showing the major disorder component only and expanded to show the complete zinc coordination polyhedra (50% displacement ellipsoids). Symmetry codes: (i) *x*, *y * − 1, *z*; (ii) *x*, *y* + 1, *z*.

Compound (I) features unusual disorder of the zincophos­phite component of the structure, in a 0.7962 (13):0.2038 (13) ratio for the major and minor components, respectively. The major component features two zinc atoms (Zn1 and Zn2), four phospho­rus atoms (P1–P4) and 12 oxygen atoms (O1–O12), the latter being parts of pseudo-pyramidal HPO_3_^2−^ hydrogenphosphite anions (Fig. 1 ▸). Both zinc ions adopt typical tetra­hedral coordination geometries to four nearby O atoms (which all bridge to an adjacent P atom) with mean Zn—O separations of 1.939 and 1.937 Å for Zn1 and Zn2, respectively. The ranges of O—Zn—O bond angles for Zn1 [101.6 (3)–126.2 (3)] and Zn2 [102.1 (3)–125.8 (3)°] seem to indicate a high degree of distortion from a regular tetra­hedral geometry for these polyhedra, but these data should be approached with caution because of the disorder of the ZnPO framework (*vide infra*). The P atoms in (I) all display their expected tetra­hedral geometries to three O atoms (two of which bridge to Zn atoms and one is ‘terminal’, hence the 1:2 Zn:P stoichiometry) and one H atom. As usual (Harrison, 2011 ▸) the H atom attached to the P atom does not show any propensity to form hydrogen bonds. The mean P—O separation for the terminal vertices (1.510 Å) is slightly shorter than the corresponding value for the bridging O atoms (1.538 Å), although there is some overlap of individual values. The O—P—O bond angles in (I) are clustered in the narrow range of 111.0 (4)–113.8 (4)° (mean = 112.5°) and are comparable to those in related structures (*e.g*. Dong *et al.*, 2015 ▸). For the oxygen atoms (O1–O12) associated with the major disorder component, the mean Zn—O—P angle is 129.6° (Table 1 ▸); four of these O atoms (O3, O6, O9 and O12) are parts of the terminal P=O vertices. The geometrical data for the minor disorder component of the chain (atoms Zn11, Zn12, P11–P14, O21–O28) are broadly similar to those of the major component, although their precision is about four to five times lower.

A striking feature of the disorder as modelled here is that atoms O1, O4, O7 and O10 are common to both major and minor components (*i.e.* they were modelled with full occupancies). These atoms are involved in the most distorted bond angles [*e.g.* O1—Zn1—O4 = 126.2 (3)°] and their mean *U*_iso_ value of 0.0191 is notably higher than the corresponding value of 0.0146 Å^2^ for the major-disorder O atoms. This may indicate that there are actually separate, adjacent, sites for the major and minor components for these O atoms but they cannot be resolved from the present data.

**Figure 2:**
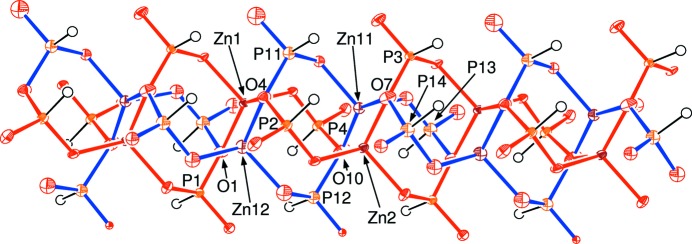
View of a fragment of a [010] zincophosphite chain in (I) showing the major (red bonds) and minor (blue bonds) disorder components with selected atoms labelled. Note that O1, O4, O7 and O10 are common to both components.

The polyhedral connectivity in (I) leads to [010] infinite anionic four-ring [Zn(HPO_3_)_2_]_*n*_^2*n*−^ chains of strictly alternating vertex-sharing ZnO_4_ and HPO_3_ groups with only translational symmetry building up the chains. Fig. 2 ▸ shows a fragment of a chain including both disorder components in which it may be seen that one can be superimposed on the other by means of a simple translation of approximately *b*/2. Each disorder component of the chain has four crystallographically unique water mol­ecules of crystallization associated with it (O1*w*–O4*w* and O11*w*–O14*w* for the major and minor disorder components, respectively) and all of them form two O—H⋯O hydrogen bonds to their adjacent chains.

**Figure 3:**
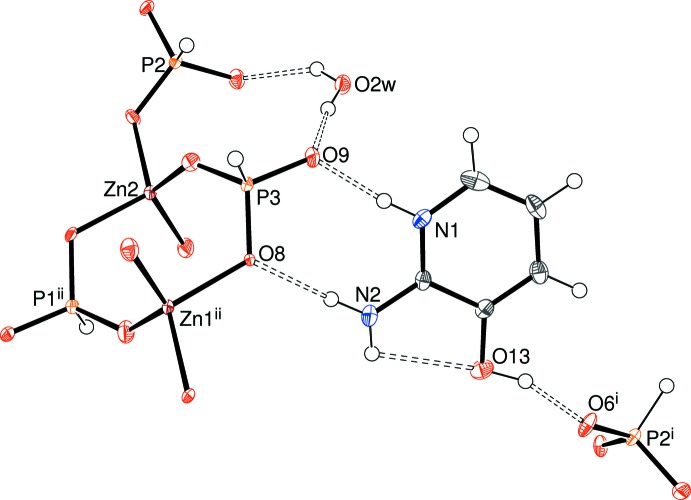
Detail of (I) showing the hydrogen-bonding inter­actions of the N1 cation with the major disorder component of the ZnPO chain.

**Figure 5:**
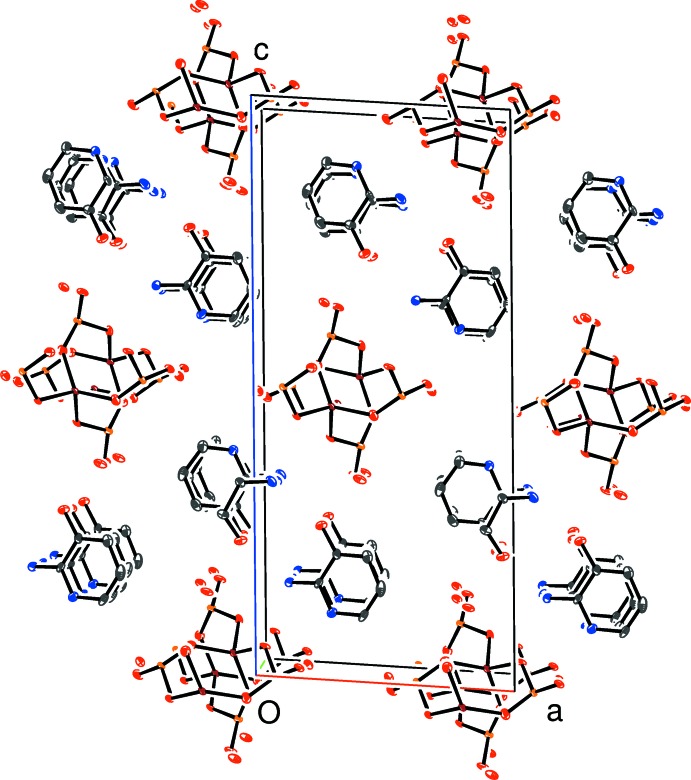
The unit-cell packing in (I) viewed down [010] with H atoms omitted for clarity.

The structure of (I) is completed by four unique, ordered, charge-balancing C_5_H_7_N_2_O^+^ cations, with each one protonated at its pyridine N atom rather than the amine group as always appears to be the case with this species (*e.g*. Stilinović & Kaitner, 2011 ▸). Each cation in (I) features an intra­molecular N—H⋯O hydrogen bond (Table 2 ▸) from the 2-amino group to the adjacent 3-hy­droxy moiety, generating an *S*(5) ring in each case. In the extended structure, each cation forms numerous N—H⋯O and O—H⋯O hydrogen bonds with chain and water O atoms from both disorder components acting as acceptors. The situation for the N1 cation is illus­trated in Figs. 3 ▸ and 4 ▸ for the major and minor disorder components of the chain, respectively. A view down [010] of the packing for (I) (Fig. 5 ▸) shows the anionic chains inter­spersed by the organic cations, which themselves form wavy (001) sheets.

**Figure 6:**
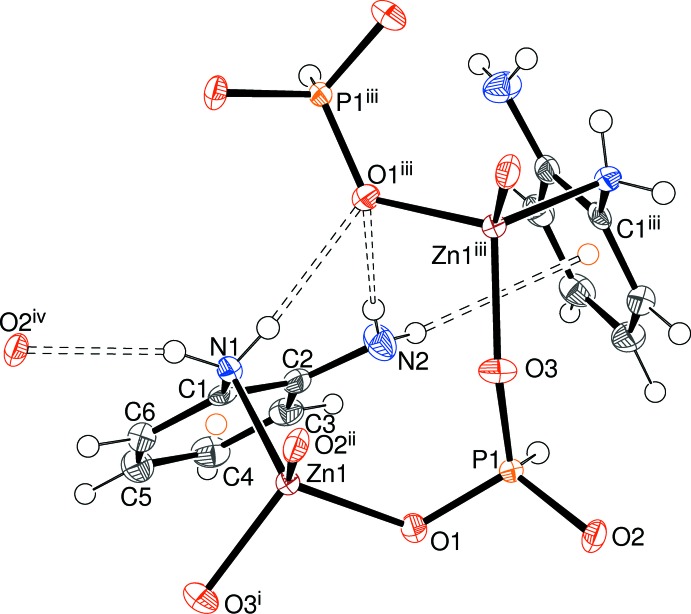
Fragment of the structure of (II) with hydrogen bonds indicated by double-dashed lines (50% displacement ellipsoids). Symmetry codes: (i) 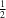 − *x*, *y*, 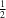 + *z*; (ii) 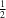 + *x*, −*y*, *z*; (iii) 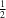 − *x*, *y*, *z* − 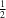; (iv) *x* + 1, *y*, *z*.

The structure of (II) consists of ZnO_3_N tetra­hedra and HPO_3_ pseudo pyramids as well as neutral 1,2-di­amino­benzene mol­ecules (Table 3 ▸, Fig. 6 ▸). The Zn—N bond, which is notably longer than the Zn—O vertices (mean = 1.935 Å) arises from a direct bond to the organic species, which could be said to be acting as a ligand rather than a (protonated) templating agent. The Zn- and P-centred polyhedra are linked by O atoms (mean Zn—O—P angle = 133.0°) and there are no terminal O atoms. This ‘3+3’ bonding mode naturally leads to the 1:1 Zn:P stoichiometry in (II).

**Figure 7:**
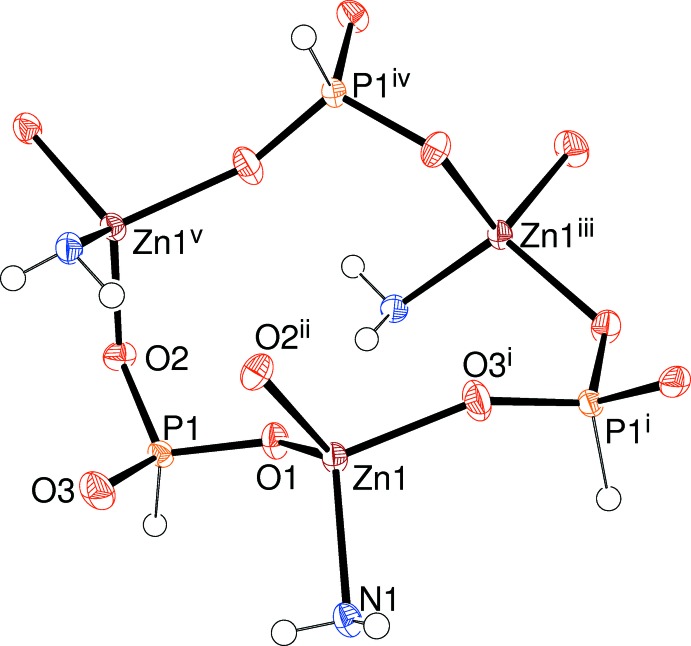
A six-ring window in (II) constructed from ZnO_3_N and HPO_3_ building units. Symmetry codes: (i) 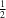 − *x*, *y*, 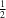 + *z*; (ii) 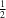 + *x*, −*y*, *z*; (iii) 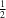 − *x*, *y*, *z * − 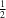; (iv) −*x*, −*y*, *z* − 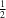; (v) *x* − 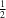, −*y*, *z*.

**Figure 8:**
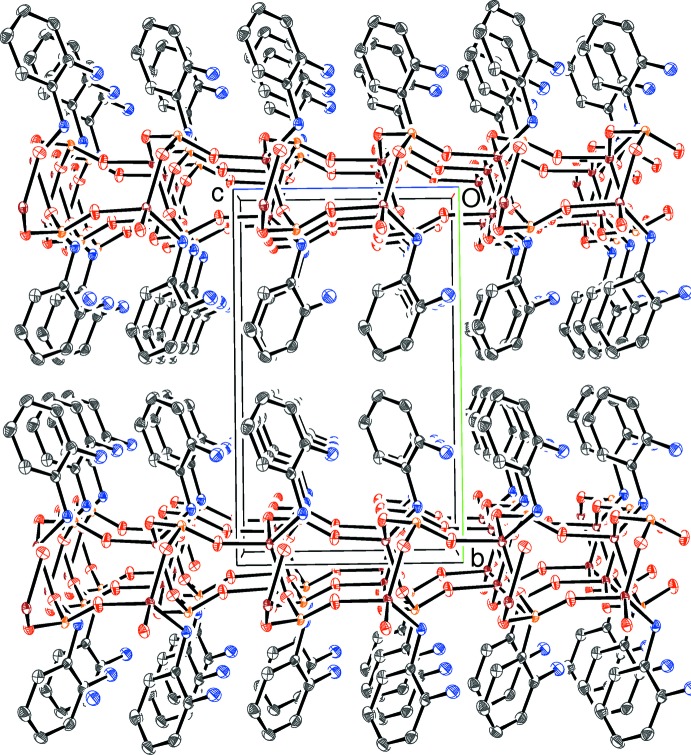
The unit-cell packing for (II) viewed down [100] with H atoms omitted for clarity.

The extended structure of (II) contains (010) sheets of strictly alternating Zn- and P-centred polyhedra incorporating very contorted six-ring windows (Fig. 7 ▸). The pendant organic mol­ecules protrude either side of the sheets (Fig. 8 ▸). The structure of (II) is consolidated by N—H⋯O hydrogen bonds, which are absolutely typical in this family of phases (Huang *et al.*, 2017 ▸) and less common N—H⋯π inter­actions (Table 4 ▸). All of these bonds are intra-sheet inter­actions and no directional inter-sheet inter­actions beyond normal van der Waals contacts could be identified, the shortest of these being H3⋯H4 (2.67 Å).

## Database survey   

So far as we are aware, no zincophosphites with either of the organic species described here have been reported previously. It may be noted that the C_6_H_7_N_2_O^+^ cation in (I) has been reported as a counter-ion with simple, discrete *M*Cl_4_^2−^ anions where *M* = Co (Koval’chukova *et al.*, 2008 ▸) and Cu (Halvorson *et al.*, 1990 ▸) and with polymeric two-dimensional copper/bromide networks (Place *et al.*, 1998 ▸). A structure containing Zn—N bonds related to (II) featuring the isomeric 1,4-di­amino­benzene species has been described (Kirkpatrick & Harrison, 2004 ▸). In this compound, the di­amine bonds to zinc atoms from both its N atoms and acts as a ‘pillar’ linking ZnPO sheets into a three-dimensional framework. A survey of of the Cambridge Structural Database (Groom *et al.*, 2016 ▸: updated to April 2018) for zinc phosphite frameworks with a directly bonded ligand/template (*i.e*. those containing a N—Zn—O—P—H fragment) revealed 21 matches.

## Synthesis and crystallization   

Compound (I) was prepared from 1.00 g ZnO, 2.00 g H_3_PO_3_ and 1.35 g 2-amino-3-hy­droxy­pyridine. These components were added to a PTFE bottle containing 20 ml of water and shaken well, to result in an off-white slurry. The bottle was sealed and placed in an oven at 353 K for 48 h and then removed to cool to room temperature. Product recovery by vacuum filtration yielded a mass of pale-brown laths of (I).

To prepare (II), 1.00 g zinc acetate, 0.74 g H_3_PO_3_, 0.99 g 1,2-di­amino­benzene and 20 ml of water were placed in a PTFE bottle and shaken well, to result in a brown slurry. The bottle was sealed and placed in an oven at 353 K for 48 h and then removed to cool to room temperature. Product recovery by vacuum filtration yielded a few colourless blocks of (II) accompanied by unidentified dark-brown sludge.

## Refinement   

Crystal data, data collection and structure refinement details are summarized in Table 5 ▸. The structure of (I) proved to be difficult to solve and refine. The systematic absences pointed to space group *P*2_1_/*n* but no chemically reasonable models could be established in this centrosymmetric space group. Lower symmetry space groups were then tried and a plausible model in *Pn* was developed, as the complex nature of the disorder of the chain became apparent. In the early stages of the refinement, site occupancies were freely varied to establish which atoms belonged to which disorder component; the occupancies for O1, O4, O7 and O10 barely varied from unity and were fixed as fully occupied. When the disorder model was becoming clear, constrained refinements of site occupancies for the major and minor disorder components (including their associated water mol­ecules of crystallization) led to refined values of 0.7962 (13):0.2038 (13). The structure of (II) was solved and refined without difficulty.

For (I), the H atoms associated with the P atoms were located in difference maps, relocated to idealized positions (P—H = 1.32 Å) and refined as riding atoms. The N- and O-bound H atoms of the cations were located in difference maps and refined as riding atoms in their as-found relative positions. Most of the water H atoms were located in difference maps and refined in a similar fashion; the remainder were placed geometrically to form reasonable hydrogen bonds and refined as riding atoms. The C-bound H atoms were placed geometrically (C—H = 0.95 Å) and refined as riding atoms. In every case, the constraint *U*_iso_(H) = 1.2*U*_eq_(carrier) was applied. The crystal of (I) chosen for data collection was found to be an inversion twin in a 0.56 (2):0.44 (2) domain ratio.

For (II), the phosphite H atom was located in a difference map, relocated to an idealized position (P—H = 1.32 Å) and refined as a riding atom. The N-bound H atoms were located in difference maps and their positions were freely refined. The C-bound H atoms were placed geometrically (C—H = 0.95 Å) and refined as riding atoms. The constraint *U*_iso_(H) = 1.2*U*_eq_(carrier) was applied to all H atoms.

## Supplementary Material

Crystal structure: contains datablock(s) I, II, global. DOI: 10.1107/S2056989018012343/cq2027sup1.cif

Structure factors: contains datablock(s) I. DOI: 10.1107/S2056989018012343/cq2027Isup2.hkl

Structure factors: contains datablock(s) II. DOI: 10.1107/S2056989018012343/cq2027IIsup3.hkl

CCDC references: 1864884, 1864883

Additional supporting information:  crystallographic information; 3D view; checkCIF report

## Figures and Tables

**Figure 4:**
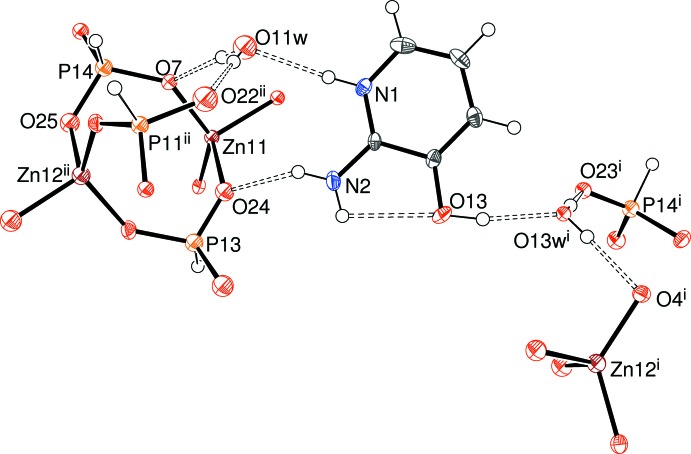
Detail of (I) showing the hydrogen-bonding inter­actions of the N1 cation with the minor disorder component of the ZnPO chain.

**Table 1:** Selected geometric parameters (Å, °) for (I)

Zn1—O1	1.922 (6)	P1—O1	1.556 (6)
Zn1—O4	1.923 (6)	P2—O6	1.494 (7)
Zn1—O11	1.953 (6)	P2—O4	1.533 (6)
Zn1—O8^i^	1.958 (6)	P2—O5	1.543 (7)
Zn2—O7	1.909 (7)	P3—O9	1.522 (8)
Zn2—O5	1.938 (6)	P3—O8	1.537 (7)
Zn2—O10	1.950 (7)	P3—O7	1.559 (6)
Zn2—O2^ii^	1.951 (7)	P4—O10	1.516 (6)
P1—O3	1.499 (7)	P4—O12	1.523 (7)
P1—O2	1.534 (7)	P4—O11	1.524 (7)
			
P1—O1—Zn1	137.6 (4)	P3—O7—Zn2	137.6 (4)
P1—O2—Zn2^i^	117.7 (4)	P3—O8—Zn1^ii^	118.4 (4)
P2—O4—Zn1	139.0 (4)	P4—O10—Zn2	140.5 (4)
P2—O5—Zn2	123.5 (4)	P4—O11—Zn1	122.8 (4)

Symmetry codes: (i) 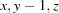; (ii) 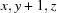.

**Table 2:** Hydrogen-bond geometry (Å, °) for (I)

*D*—H⋯*A*	*D*—H	H⋯*A*	*D*⋯*A*	*D*—H⋯*A*
C5—H5⋯O1*W* ^iii^	0.95	2.60	3.539 (13)	169
N1—H1*N*⋯O9	0.88	1.73	2.595 (10)	168
N2—H2*N*⋯O16^iv^	0.85	2.31	3.060 (10)	147
N2—H3*N*⋯O8	0.87	2.10	2.926 (9)	157
O13—H1*O*⋯O6^iv^	0.96	1.58	2.488 (9)	156
C8—H8⋯O3*W* ^iv^	0.95	2.55	3.225 (13)	128
C9—H9⋯O4*W* ^v^	0.95	2.61	3.562 (12)	178
C10—H10⋯O3^iii^	0.95	2.54	3.486 (14)	171
N3—H4*N*⋯O2*W*	0.88	1.93	2.771 (10)	160
N4—H5*N*⋯O15^iv^	0.86	2.22	2.959 (10)	144
N4—H6*N*⋯O11	0.94	2.01	2.853 (9)	148
O14—H2*O*⋯O3*W* ^iv^	0.94	1.78	2.656 (9)	154
C14—H14⋯O3*W* ^vi^	0.95	2.54	3.490 (12)	175
C15—H15⋯O9^vi^	0.95	2.50	3.442 (14)	172
N5—H7*N*⋯O1*W*	0.88	1.91	2.756 (9)	162
N6—H8*N*⋯O13^vii^	0.91	2.19	3.019 (9)	151
N6—H9*N*⋯O5	0.91	1.97	2.824 (10)	156
O15—H3*O*⋯O12^vii^	0.95	1.57	2.474 (9)	157
C18—H18⋯O4*W* ^vii^	0.95	2.47	3.134 (12)	127
C20—H20⋯O2*W* ^vi^	0.95	2.59	3.508 (13)	163
N7—H10*N*⋯O3	0.88	1.73	2.595 (10)	169
N8—H11*N*⋯O14^viii^	0.91	2.30	3.090 (9)	145
N8—H12*N*⋯O2	0.85	2.21	3.012 (9)	157
O16—H4*O*⋯O4*W* ^vii^	0.92	1.72	2.611 (10)	161
O1*W*—H1*W*⋯O10	0.72	2.13	2.787 (9)	152
O1*W*—H2*W*⋯O3	0.80	1.94	2.732 (8)	171
O2*W*—H3*W*⋯O4	0.84	2.04	2.785 (9)	147
O2*W*—H4*W*⋯O9	0.89	1.83	2.711 (9)	168
O3*W*—H5*W*⋯O6^ii^	0.91	1.79	2.694 (9)	177
O3*W*—H6*W*⋯O7	0.84	1.96	2.797 (9)	180
O4*W*—H7*W*⋯O12	0.93	1.77	2.694 (9)	172
O4*W*—H8*W*⋯O1^ii^	0.80	2.03	2.760 (9)	150
O11*W*—H11*W*⋯O22^ii^	0.82	1.90	2.72 (4)	178
O11*W*—H12*W*⋯O7	0.78	1.87	2.66 (4)	178
O12*W*—H13*W*⋯O1	0.80	1.84	2.65 (3)	179
O12*W*—H14*W*⋯O21^i^	0.83	1.98	2.81 (3)	179
O13*W*—H15*W*⋯O4	0.80	1.88	2.68 (3)	178
O13*W*—H16*W*⋯O23	0.83	1.91	2.74 (4)	177
O14*W*—H17*W*⋯O10	0.82	1.92	2.73 (4)	176
O14*W*—H18*W*⋯O27^i^	0.80	1.92	2.72 (4)	176

Symmetry codes: (i) 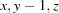; (ii) 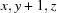; (iii) 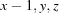; (iv) 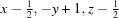; (v) 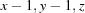; (vi) 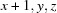; (vii) 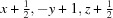; (viii) 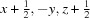.

**Table 3:** Selected geometric parameters (Å, °) for (II)

Zn1—O3^i^	1.918 (2)	P1—O3	1.501 (2)
Zn1—O2^ii^	1.9425 (17)	P1—O1	1.529 (3)
Zn1—O1	1.9445 (16)	P1—O2	1.5299 (19)
Zn1—N1	2.056 (3)		
			
P1—O1—Zn1	123.02 (15)	P1—O3—Zn1^iv^	155.43 (13)
P1—O2—Zn1^iii^	120.64 (10)		

Symmetry codes: (i) 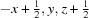; (ii) 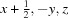; (iii) 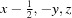; (iv) 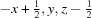.

**Table 4:** Hydrogen-bond geometry (Å, °) for (II)

*D*—H⋯*A*	*D*—H	H⋯*A*	*D*⋯*A*	*D*—H⋯*A*
N1—H1*N*⋯O2^v^	0.85 (3)	2.15 (4)	2.966 (3)	161 (3)
N1—H2*N*⋯O1^iv^	0.80 (3)	2.22 (4)	3.011 (4)	171 (3)
N2—H4*N*⋯O1^iv^	0.79 (4)	2.21 (4)	2.998 (4)	174 (4)
N2—H3*N*⋯*Cg* ^iv^	0.82 (4)	2.80 (4)	3.400 (3)	132 (3)

Symmetry codes: (iv) 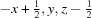; (v) 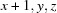.

**Table 5:** Experimental details

	(I)	(II)
Crystal data		
Chemical formula	(C_5_H_7_N_2_O)[Zn(HPO_3_)_2_]·2H_2_O	[Zn(HPO_3_)(C_6_H_8_N_2_)]
*M*_r_	483.61	253.49
Crystal system, space group	Monoclinic, *P* *n*	Orthorhombic, *P* *c* *a*2_1_
Temperature (K)	100	173
*a*, *b*, *c* (Å)	10.5172 (3), 7.4210 (2), 23.5592 (5)	8.0419 (2), 13.5008 (4), 8.1307 (2)
α, β, γ (°)	90, 93.861 (2), 90	90, 90, 90
*V* (Å^3^)	1834.58 (8)	882.77 (4)
*Z*	4	4
Radiation type	Mo *K*α	Mo *K*α
μ (mm^−1^)	1.57	2.94
Crystal size (mm)	0.20 × 0.05 × 0.04	0.27 × 0.10 × 0.02

Data collection		
Diffractometer	Rigaku XtaLAB AFC12 (RCD3): Kappa single CCD	Rigaku XtaLAB P200 HPC
Absorption correction	Gaussian (*CrysAlis PRO*; Rigaku OD, 2017 ▸)	Multi-scan (*CrysAlis PRO*; Rigaku OD, 2017 ▸)
*T*_min_, *T*_max_	0.653, 1.000	0.731, 1.000
No. of measured, independent and observed [*I* > 2σ(*I*)] reflections	30281, 8399, 7166	11004, 2038, 1952
*R*_int_	0.070	0.044
(sin θ/λ)_max_ (Å^−1^)	0.649	0.685

Refinement		
*R*[*F* ^2^ > 2σ(*F* ^2^)], *wR*(*F* ^2^), *S*	0.046, 0.121, 1.05	0.022, 0.051, 1.04
No. of reflections	8399	2038
No. of parameters	561	131
No. of restraints	116	1
H-atom treatment	H-atom parameters constrained	H atoms treated by a mixture of independent and constrained refinement
Δρ_max_, Δρ_min_ (e Å^−3^)	0.73, −0.93	0.71, −0.27
Absolute structure	Refined as an inversion twin.	Flack (1983 ▸) parameter
Absolute structure parameter	0.44 (2)	0.016 (14)

Computer programs: *CrysAlis PRO* (Rigaku OD, 2017 ▸), *CrysAlis PRO* (Rigaku, 2017 ▸), *SHELXS97* (Sheldrick, 2008 ▸), *SHELXL2014* (Sheldrick, 2015 ▸), *ORTEP-3 for Windows* (Farrugia, 2012 ▸) and *publCIF* (Westrip, 2010 ▸).

## supplementary crystallographic information

### catena-Poly[bis(2-amino-3-hydroxypyridinium) [zinc-di-µ-phosphonato] dihydrate] (I) Crystal data

**Table:**

(C_5_H_7_N_2_O)[Zn(HPO_3_)_2_]·2H_2_O	*F*(000) = 992
*M**_r_* = 483.61	*D*_x_ = 1.751 Mg m^−^^3^
Monoclinic, *P**n*	Mo *K*α radiation, λ = 0.71073 Å
*a* = 10.5172 (3) Å	Cell parameters from 11223 reflections
*b* = 7.4210 (2) Å	θ = 3.4–30.0°
*c* = 23.5592 (5) Å	µ = 1.57 mm^−^^1^
β = 93.861 (2)°	*T* = 100 K
*V* = 1834.58 (8) Å^3^	Lath, pale brown
*Z* = 4	0.20 × 0.05 × 0.04 mm

### catena-Poly[bis(2-amino-3-hydroxypyridinium) [zinc-di-µ-phosphonato] dihydrate] (I) Data collection

**Table:**

Rigaku XtaLAB AFC12 (RCD3): Kappa single CCD diffractometer	7166 reflections with *I* > 2σ(*I*)
ω scans	*R*_int_ = 0.070
Absorption correction: gaussian (CrysAlis PRO; Rigaku OD, 2017)	θ_max_ = 27.5°, θ_min_ = 2.9°
*T*_min_ = 0.653, *T*_max_ = 1.000	*h* = −13→13
30281 measured reflections	*k* = −9→9
8399 independent reflections	*l* = −30→30

### catena-Poly[bis(2-amino-3-hydroxypyridinium) [zinc-di-µ-phosphonato] dihydrate] (I) Refinement

**Table:**

Refinement on *F*^2^	Secondary atom site location: difference Fourier map
Least-squares matrix: full	Hydrogen site location: mixed
*R*[*F*^2^ > 2σ(*F*^2^)] = 0.046	H-atom parameters constrained
*wR*(*F*^2^) = 0.121	*w* = 1/[σ^2^(*F*_o_^2^) + (0.0553*P*)^2^ + 0.7374*P*] where *P* = (*F*_o_^2^ + 2*F*_c_^2^)/3
*S* = 1.05	(Δ/σ)_max_ = 0.001
8399 reflections	Δρ_max_ = 0.73 e Å^−^^3^
561 parameters	Δρ_min_ = −0.93 e Å^−^^3^
116 restraints	Absolute structure: Refined as an inversion twin.
Primary atom site location: structure-invariant direct methods	Absolute structure parameter: 0.44 (2)

### catena-Poly[bis(2-amino-3-hydroxypyridinium) [zinc-di-µ-phosphonato] dihydrate] (I) Special details

**Table:**

Geometry. All esds (except the esd in the dihedral angle between two l.s. planes) are estimated using the full covariance matrix. The cell esds are taken into account individually in the estimation of esds in distances, angles and torsion angles; correlations between esds in cell parameters are only used when they are defined by crystal symmetry. An approximate (isotropic) treatment of cell esds is used for estimating esds involving l.s. planes.
Refinement. Refined as a 2-component inversion twin

### catena-Poly[bis(2-amino-3-hydroxypyridinium) [zinc-di-µ-phosphonato] dihydrate] (I) Fractional atomic coordinates and isotropic or equivalent isotropic displacement parameters (Å^2^)

**Table:**

	*x*	*y*	*z*	*U*_iso_*/*U*_eq_	Occ. (<1)
Zn1	0.29788 (9)	0.12391 (12)	0.47099 (5)	0.0097 (2)	0.7962 (13)
Zn2	0.39776 (9)	0.63750 (12)	0.52509 (5)	0.0107 (2)	0.7962 (13)
P1	0.5742 (2)	−0.0631 (3)	0.49814 (11)	0.0100 (5)	0.7962 (13)
H1P	0.6345	−0.1706	0.4643	0.012*	0.7962 (13)
P2	0.2957 (2)	0.3226 (3)	0.59341 (11)	0.0117 (5)	0.7962 (13)
H2P	0.2057	0.4351	0.6074	0.014*	0.7962 (13)
P3	0.1214 (2)	0.8237 (3)	0.50040 (10)	0.0096 (5)	0.7962 (13)
H3P	0.0635	0.9329	0.5346	0.012*	0.7962 (13)
P4	0.4029 (3)	0.4338 (3)	0.40242 (11)	0.0121 (5)	0.7962 (13)
H4P	0.4949	0.3204	0.3916	0.015*	0.7962 (13)
O1	0.4630 (6)	0.0229 (7)	0.4606 (3)	0.0187 (12)	
O2	0.5283 (7)	−0.1830 (8)	0.5457 (3)	0.0130 (15)	0.7962 (13)
O3	0.6701 (7)	0.0742 (9)	0.5199 (3)	0.0128 (14)	0.7962 (13)
O4	0.2409 (6)	0.2209 (7)	0.5406 (3)	0.0166 (12)	
O5	0.4101 (6)	0.4405 (8)	0.5792 (3)	0.0141 (15)	0.7962 (13)
O6	0.3238 (8)	0.2047 (9)	0.6441 (3)	0.0173 (15)	0.7962 (13)
O7	0.2333 (6)	0.7341 (7)	0.5368 (3)	0.0195 (12)	
O8	0.1677 (7)	0.9409 (8)	0.4521 (3)	0.0108 (14)	0.7962 (13)
O9	0.0226 (7)	0.6864 (10)	0.4784 (3)	0.0187 (16)	0.7962 (13)
O10	0.4529 (6)	0.5428 (7)	0.4536 (3)	0.0215 (13)	
O11	0.2885 (7)	0.3175 (8)	0.4147 (3)	0.0122 (14)	0.7962 (13)
O12	0.3790 (8)	0.5469 (9)	0.3488 (3)	0.0180 (16)	0.7962 (13)
C1	−0.0430 (9)	0.7452 (9)	0.3345 (4)	0.0127 (15)	
C2	−0.1081 (8)	0.7173 (10)	0.2791 (3)	0.0153 (16)	
C3	−0.2282 (10)	0.6467 (10)	0.2765 (4)	0.0197 (18)	
H3	−0.2730	0.6291	0.2405	0.024*	
C4	−0.2865 (9)	0.5996 (12)	0.3264 (4)	0.0209 (19)	
H4	−0.3712	0.5541	0.3247	0.025*	
C5	−0.2178 (11)	0.6210 (10)	0.3778 (5)	0.023 (2)	
H5	−0.2530	0.5846	0.4121	0.028*	
N1	−0.1006 (7)	0.6937 (8)	0.3792 (3)	0.0155 (14)	
H1N	−0.0595	0.7076	0.4127	0.019*	
N2	0.0727 (7)	0.8217 (9)	0.3387 (3)	0.0192 (15)	
H2N	0.1103	0.8436	0.3085	0.023*	
H3N	0.1172	0.8327	0.3713	0.023*	
O13	−0.0441 (6)	0.7701 (8)	0.2354 (3)	0.0205 (13)	
H1O	−0.1011	0.7477	0.2025	0.025*	
C6	−0.0411 (8)	0.2483 (9)	0.3353 (4)	0.0132 (15)	
C7	−0.1160 (8)	0.2107 (10)	0.2836 (4)	0.0154 (16)	
C8	−0.2329 (10)	0.1395 (10)	0.2858 (5)	0.0214 (19)	
H8	−0.2832	0.1163	0.2516	0.026*	
C9	−0.2817 (9)	0.0987 (12)	0.3391 (4)	0.0219 (19)	
H9	−0.3647	0.0495	0.3411	0.026*	
C10	−0.2057 (10)	0.1323 (9)	0.3877 (5)	0.020 (2)	
H10	−0.2348	0.1036	0.4239	0.023*	
N3	−0.0902 (7)	0.2056 (8)	0.3837 (3)	0.0147 (14)	
H4N	−0.0439	0.2268	0.4155	0.018*	
N4	0.0743 (7)	0.3240 (9)	0.3337 (3)	0.0176 (15)	
H5N	0.1026	0.3503	0.3014	0.021*	
H6N	0.1196	0.3271	0.3696	0.021*	
O14	−0.0615 (6)	0.2569 (8)	0.2359 (3)	0.0187 (13)	
H2O	−0.1066	0.2487	0.2002	0.022*	
C11	0.7386 (9)	0.5063 (9)	0.6604 (3)	0.0132 (15)	
C12	0.8096 (8)	0.5368 (10)	0.7127 (4)	0.0137 (15)	
C13	0.9285 (10)	0.6141 (10)	0.7120 (5)	0.0193 (18)	
H13	0.9764	0.6387	0.7467	0.023*	
C14	0.9794 (10)	0.6566 (12)	0.6599 (4)	0.024 (2)	
H14	1.0616	0.7096	0.6598	0.029*	
C15	0.9143 (10)	0.6237 (9)	0.6116 (5)	0.021 (2)	
H15	0.9509	0.6487	0.5767	0.025*	
N5	0.7913 (7)	0.5519 (8)	0.6112 (3)	0.0153 (14)	
H7N	0.7471	0.5361	0.5786	0.018*	
N6	0.6241 (7)	0.4317 (10)	0.6582 (3)	0.0175 (15)	
H8N	0.5904	0.3944	0.6910	0.021*	
H9N	0.5721	0.4240	0.6261	0.021*	
O15	0.7456 (6)	0.4867 (8)	0.7589 (3)	0.0200 (13)	
H3O	0.8041	0.5036	0.7911	0.024*	
C16	0.7461 (8)	0.0065 (9)	0.6661 (4)	0.0127 (15)	
C17	0.8178 (8)	0.0341 (10)	0.7183 (4)	0.0149 (16)	
C18	0.9372 (9)	0.1088 (10)	0.7185 (4)	0.0189 (18)	
H18	0.9857	0.1277	0.7535	0.023*	
C19	0.9884 (10)	0.1580 (12)	0.6663 (5)	0.024 (2)	
H19	1.0712	0.2092	0.6663	0.029*	
C20	0.9206 (10)	0.1322 (9)	0.6180 (5)	0.0185 (19)	
H20	0.9553	0.1627	0.5830	0.022*	
N7	0.7986 (7)	0.0608 (8)	0.6178 (3)	0.0179 (15)	
H10N	0.7535	0.0502	0.5851	0.021*	
N8	0.6313 (7)	−0.0675 (9)	0.6625 (3)	0.0161 (14)	
H11N	0.5974	−0.0978	0.6959	0.019*	
H12N	0.5858	−0.0779	0.6314	0.019*	
O16	0.7579 (6)	−0.0175 (8)	0.7652 (3)	0.0193 (13)	
H4O	0.8095	−0.0032	0.7982	0.023*	
Zn11	0.3031 (7)	0.6147 (5)	0.4721 (3)	0.0135 (10)*	0.2038 (13)
Zn12	0.4040 (7)	0.1296 (5)	0.5270 (3)	0.0187 (12)*	0.2038 (13)
P11	0.1234 (12)	0.3165 (13)	0.4996 (5)	0.016 (2)*	0.2038 (13)
H11P	0.0600	0.4218	0.5330	0.019*	0.2038 (13)
P12	0.5783 (12)	0.4298 (14)	0.4968 (6)	0.016 (2)*	0.2038 (13)
H12P	0.6349	0.3214	0.4620	0.020*	0.2038 (13)
P13	0.4039 (13)	0.9269 (15)	0.4043 (6)	0.019 (2)*	0.2038 (13)
H13P	0.4927	0.8130	0.3899	0.022*	0.2038 (13)
P14	0.2970 (12)	0.8186 (14)	0.5952 (6)	0.018 (2)*	0.2038 (13)
H14P	0.2128	0.9396	0.6095	0.021*	0.2038 (13)
O21	0.675 (2)	0.558 (3)	0.5173 (10)	0.006 (5)*	0.2038 (13)
O22	0.024 (3)	0.171 (4)	0.4733 (14)	0.032 (8)*	0.2038 (13)
O23	0.318 (3)	0.712 (3)	0.6457 (13)	0.016 (6)*	0.2038 (13)
O24	0.298 (3)	0.815 (4)	0.4172 (13)	0.021 (6)*	0.2038 (13)
O25	0.416 (3)	0.935 (4)	0.5815 (13)	0.022 (7)*	0.2038 (13)
O26	0.535 (3)	0.311 (4)	0.5443 (14)	0.025 (7)*	0.2038 (13)
O27	0.375 (3)	1.048 (4)	0.3498 (15)	0.026 (7)*	0.2038 (13)
O28	0.170 (3)	0.437 (3)	0.4515 (12)	0.013 (6)*	0.2038 (13)
O1W	0.6860 (7)	0.4392 (8)	0.5070 (3)	0.0134 (14)	0.7962 (13)
H1W	0.6406	0.4900	0.4889	0.016*	0.7962 (13)
H2W	0.6747	0.3343	0.5127	0.016*	0.7962 (13)
O2W	0.0054 (7)	0.3238 (9)	0.4897 (3)	0.0155 (15)	0.7962 (13)
H3W	0.0564	0.2613	0.5103	0.019*	0.7962 (13)
H4W	0.0227	0.4412	0.4873	0.019*	0.7962 (13)
O3W	0.2802 (7)	0.8476 (9)	0.6495 (3)	0.0145 (15)	0.7962 (13)
H5W	0.2959	0.9675	0.6465	0.017*	0.7962 (13)
H6W	0.2657	0.8138	0.6159	0.017*	0.7962 (13)
O4W	0.4104 (7)	0.9072 (10)	0.3501 (3)	0.0186 (16)	0.7962 (13)
H7W	0.4055	0.7825	0.3475	0.022*	0.7962 (13)
H8W	0.4376	0.9703	0.3758	0.022*	0.7962 (13)
O11W	0.001 (3)	0.811 (4)	0.4927 (14)	0.032 (8)*	0.2038 (13)
H11W	0.0064	0.9202	0.4871	0.038*	0.2038 (13)
H12W	0.0688	0.7889	0.5063	0.038*	0.2038 (13)
O12W	0.688 (3)	−0.064 (3)	0.5100 (11)	0.016 (6)*	0.2038 (13)
H13W	0.6193	−0.0367	0.4955	0.019*	0.2038 (13)
H14W	0.6829	−0.1755	0.5124	0.019*	0.2038 (13)
O13W	0.280 (3)	0.347 (4)	0.6471 (12)	0.012 (6)*	0.2038 (13)
H15W	0.2663	0.3098	0.6153	0.014*	0.2038 (13)
H16W	0.2890	0.4585	0.6469	0.014*	0.2038 (13)
O14W	0.410 (3)	0.411 (5)	0.3459 (15)	0.031 (7)*	0.2038 (13)
H17W	0.4193	0.4521	0.3779	0.037*	0.2038 (13)
H18W	0.3956	0.3043	0.3470	0.037*	0.2038 (13)

### catena-Poly[bis(2-amino-3-hydroxypyridinium) [zinc-di-µ-phosphonato] dihydrate] (I) Atomic displacement parameters (Å^2^)

**Table:**

	*U*^11^	*U*^22^	*U*^33^	*U*^12^	*U*^13^	*U*^23^
Zn1	0.0089 (5)	0.0115 (4)	0.0084 (4)	−0.0025 (3)	−0.0025 (3)	0.0013 (3)
Zn2	0.0104 (5)	0.0119 (4)	0.0096 (5)	−0.0026 (3)	−0.0019 (3)	0.0012 (3)
P1	0.0089 (10)	0.0114 (10)	0.0095 (9)	−0.0009 (7)	−0.0006 (7)	0.0006 (7)
P2	0.0133 (11)	0.0133 (10)	0.0080 (9)	−0.0032 (8)	−0.0013 (7)	0.0002 (7)
P3	0.0087 (10)	0.0119 (9)	0.0080 (9)	−0.0009 (7)	−0.0016 (7)	0.0009 (7)
P4	0.0134 (11)	0.0139 (10)	0.0088 (9)	−0.0032 (8)	−0.0012 (8)	0.0009 (7)
O1	0.014 (3)	0.024 (3)	0.018 (3)	−0.001 (2)	−0.002 (2)	−0.003 (2)
O2	0.019 (3)	0.013 (3)	0.006 (3)	−0.006 (2)	−0.004 (2)	0.0006 (19)
O3	0.015 (3)	0.013 (3)	0.010 (3)	−0.003 (2)	−0.004 (2)	0.004 (2)
O4	0.015 (3)	0.017 (2)	0.017 (3)	−0.001 (2)	0.000 (2)	−0.005 (2)
O5	0.009 (3)	0.015 (3)	0.018 (3)	−0.004 (2)	−0.002 (2)	0.005 (2)
O6	0.023 (3)	0.019 (3)	0.009 (3)	−0.009 (3)	−0.005 (2)	0.005 (2)
O7	0.018 (3)	0.027 (3)	0.013 (3)	−0.005 (2)	0.001 (2)	0.000 (2)
O8	0.010 (3)	0.013 (3)	0.008 (2)	−0.006 (2)	−0.003 (2)	0.0034 (19)
O9	0.019 (3)	0.020 (3)	0.016 (3)	−0.009 (3)	−0.005 (2)	0.000 (2)
O10	0.021 (3)	0.031 (3)	0.013 (3)	−0.009 (2)	0.002 (2)	−0.007 (2)
O11	0.012 (3)	0.015 (3)	0.009 (3)	−0.004 (2)	−0.005 (2)	0.0054 (19)
O12	0.026 (4)	0.017 (3)	0.010 (3)	−0.005 (3)	−0.007 (2)	0.004 (2)
C1	0.015 (4)	0.009 (3)	0.014 (3)	0.001 (3)	−0.002 (3)	−0.004 (3)
C2	0.021 (4)	0.010 (3)	0.014 (4)	0.000 (3)	−0.002 (3)	−0.001 (3)
C3	0.019 (4)	0.017 (4)	0.022 (4)	−0.003 (3)	−0.002 (3)	−0.005 (3)
C4	0.012 (4)	0.017 (4)	0.034 (5)	−0.001 (3)	0.002 (3)	−0.001 (3)
C5	0.024 (5)	0.015 (4)	0.032 (5)	0.003 (3)	0.014 (4)	0.002 (3)
N1	0.018 (3)	0.015 (3)	0.012 (3)	0.004 (3)	−0.001 (3)	0.001 (2)
N2	0.018 (4)	0.023 (3)	0.017 (3)	−0.004 (3)	−0.005 (3)	0.000 (3)
O13	0.026 (3)	0.024 (3)	0.012 (3)	0.001 (2)	0.005 (2)	−0.002 (2)
C6	0.009 (4)	0.014 (3)	0.016 (4)	0.001 (3)	−0.005 (3)	−0.001 (3)
C7	0.020 (4)	0.012 (3)	0.014 (4)	0.000 (3)	−0.001 (3)	0.001 (3)
C8	0.019 (4)	0.020 (4)	0.025 (4)	0.002 (3)	−0.006 (3)	−0.006 (3)
C9	0.017 (5)	0.015 (3)	0.035 (5)	−0.002 (3)	0.008 (4)	−0.001 (3)
C10	0.017 (4)	0.017 (4)	0.025 (4)	0.001 (3)	0.001 (3)	0.007 (3)
N3	0.014 (3)	0.014 (3)	0.016 (3)	0.004 (2)	0.001 (3)	0.005 (2)
N4	0.017 (4)	0.022 (3)	0.014 (3)	−0.001 (3)	−0.003 (3)	0.000 (3)
O14	0.021 (3)	0.023 (3)	0.014 (3)	0.001 (2)	0.005 (2)	−0.004 (2)
C11	0.018 (4)	0.010 (3)	0.012 (4)	0.003 (3)	0.006 (3)	0.003 (3)
C12	0.012 (4)	0.016 (4)	0.014 (4)	0.008 (3)	0.004 (3)	0.000 (3)
C13	0.016 (4)	0.013 (4)	0.029 (5)	0.004 (3)	−0.001 (3)	−0.008 (3)
C14	0.017 (5)	0.014 (4)	0.043 (5)	−0.005 (3)	0.010 (4)	−0.004 (4)
C15	0.021 (4)	0.013 (4)	0.031 (5)	−0.001 (3)	0.016 (4)	−0.001 (3)
N5	0.019 (3)	0.018 (3)	0.010 (3)	0.001 (3)	0.008 (3)	−0.002 (2)
N6	0.012 (3)	0.026 (3)	0.015 (3)	−0.004 (3)	0.005 (3)	0.001 (3)
O15	0.023 (3)	0.023 (3)	0.014 (3)	0.002 (2)	0.003 (2)	0.003 (2)
C16	0.012 (4)	0.011 (3)	0.017 (4)	0.003 (3)	0.007 (3)	0.003 (3)
C17	0.011 (4)	0.017 (4)	0.017 (4)	0.006 (3)	0.001 (3)	−0.002 (3)
C18	0.017 (4)	0.014 (4)	0.025 (4)	−0.001 (3)	−0.002 (3)	−0.005 (3)
C19	0.014 (4)	0.017 (4)	0.042 (5)	−0.004 (3)	0.003 (3)	−0.004 (4)
C20	0.015 (4)	0.016 (4)	0.025 (4)	−0.001 (3)	0.011 (3)	0.005 (3)
N7	0.016 (3)	0.022 (3)	0.016 (3)	0.001 (3)	0.004 (3)	0.005 (3)
N8	0.014 (3)	0.020 (3)	0.014 (3)	−0.008 (3)	0.002 (3)	0.003 (3)
O16	0.021 (3)	0.024 (3)	0.013 (3)	0.000 (2)	0.004 (2)	0.004 (2)
O1W	0.014 (3)	0.010 (3)	0.015 (3)	0.000 (2)	−0.004 (2)	0.001 (2)
O2W	0.014 (3)	0.017 (3)	0.016 (3)	0.000 (2)	−0.003 (2)	0.001 (2)
O3W	0.017 (3)	0.015 (3)	0.011 (3)	−0.004 (2)	−0.004 (2)	0.001 (2)
O4W	0.027 (4)	0.016 (3)	0.012 (3)	0.000 (3)	−0.006 (2)	−0.003 (2)

### catena-Poly[bis(2-amino-3-hydroxypyridinium) [zinc-di-µ-phosphonato] dihydrate] (I) Geometric parameters (Å, º)

**Table:**

Zn1—O1	1.922 (6)	C11—N6	1.322 (11)
Zn1—O4	1.923 (6)	C11—N5	1.360 (11)
Zn1—O11	1.953 (6)	C11—C12	1.415 (11)
Zn1—O8^i^	1.958 (6)	C12—O15	1.369 (10)
Zn2—O7	1.909 (7)	C12—C13	1.378 (13)
Zn2—O5	1.938 (6)	C13—C14	1.406 (15)
Zn2—O10	1.950 (7)	C13—H13	0.9500
Zn2—O2^ii^	1.951 (7)	C14—C15	1.311 (15)
P1—O3	1.499 (7)	C14—H14	0.9500
P1—O2	1.534 (7)	C15—N5	1.399 (13)
P1—O1	1.556 (6)	C15—H15	0.9500
P1—H1P	1.3200	N5—H7N	0.8800
P2—O6	1.494 (7)	N6—H8N	0.9143
P2—O4	1.533 (6)	N6—H9N	0.9058
P2—O5	1.543 (7)	O15—H3O	0.9530
P2—H2P	1.3200	C16—N8	1.324 (11)
P3—O9	1.522 (8)	C16—N7	1.360 (11)
P3—O8	1.537 (7)	C16—C17	1.412 (11)
P3—O7	1.559 (6)	C17—O16	1.363 (10)
P3—H3P	1.3200	C17—C18	1.373 (13)
P4—O10	1.516 (6)	C18—C19	1.423 (15)
P4—O12	1.523 (7)	C18—H18	0.9500
P4—O11	1.524 (7)	C19—C20	1.317 (15)
P4—H4P	1.3200	C19—H19	0.9500
O1—P13^i^	1.594 (14)	C20—N7	1.388 (12)
O1—Zn12	1.895 (10)	C20—H20	0.9500
O2—Zn2^i^	1.951 (7)	N7—H10N	0.8800
O4—P11	1.674 (12)	N8—H11N	0.9147
O4—Zn12	1.891 (9)	N8—H12N	0.8500
O7—P14	1.615 (13)	O16—H4O	0.9249
O7—Zn11	1.950 (9)	Zn11—O28	1.96 (3)
O8—Zn1^ii^	1.958 (6)	Zn11—O24	1.97 (3)
O10—Zn11	1.746 (9)	Zn12—O25^i^	1.93 (3)
O10—P12	1.816 (13)	Zn12—O26	1.94 (3)
C1—N1	1.306 (11)	P11—O28	1.55 (3)
C1—N2	1.341 (12)	P11—O22	1.60 (3)
C1—C2	1.449 (11)	P11—H11P	1.3200
C2—O13	1.326 (10)	P12—O21	1.45 (3)
C2—C3	1.365 (13)	P12—O26	1.52 (3)
C3—C4	1.406 (14)	P12—H12P	1.3200
C3—H3	0.9500	P13—O24	1.44 (3)
C4—C5	1.377 (15)	P13—O27	1.58 (3)
C4—H4	0.9500	P13—O1^ii^	1.594 (14)
C5—N1	1.345 (13)	P13—H13P	1.3200
C5—H5	0.9500	P14—O23	1.43 (3)
N1—H1N	0.8800	P14—O25	1.58 (3)
N2—H2N	0.8530	P14—H14P	1.3200
N2—H3N	0.8749	O25—Zn12^ii^	1.93 (3)
O13—H1O	0.9625	O1W—H1W	0.7246
C6—N3	1.322 (11)	O1W—H2W	0.8001
C6—N4	1.340 (11)	O2W—H3W	0.8384
C6—C7	1.434 (11)	O2W—H4W	0.8930
C7—O14	1.340 (10)	O3W—H5W	0.9093
C7—C8	1.342 (13)	O3W—H6W	0.8355
C8—C9	1.421 (14)	O4W—H7W	0.9286
C8—H8	0.9500	O4W—H8W	0.8023
C9—C10	1.373 (14)	O11W—H11W	0.8200
C9—H9	0.9500	O11W—H12W	0.7846
C10—N3	1.339 (12)	O12W—H13W	0.8024
C10—H10	0.9500	O12W—H14W	0.8329
N3—H4N	0.8800	O13W—H15W	0.8035
N4—H5N	0.8578	O13W—H16W	0.8307
N4—H6N	0.9428	O14W—H17W	0.8153
O14—H2O	0.9391	O14W—H18W	0.8034
			
O1—Zn1—O4	126.2 (3)	N6—C11—N5	119.6 (8)
O1—Zn1—O11	101.8 (3)	N6—C11—C12	121.7 (8)
O4—Zn1—O11	107.4 (3)	N5—C11—C12	118.7 (8)
O1—Zn1—O8^i^	108.9 (3)	O15—C12—C13	128.2 (8)
O4—Zn1—O8^i^	101.6 (3)	O15—C12—C11	112.9 (7)
O11—Zn1—O8^i^	110.7 (3)	C13—C12—C11	118.8 (8)
O7—Zn2—O5	102.1 (3)	C12—C13—C14	120.2 (10)
O7—Zn2—O10	125.8 (3)	C12—C13—H13	119.9
O5—Zn2—O10	106.6 (3)	C14—C13—H13	119.9
O7—Zn2—O2^ii^	109.7 (3)	C15—C14—C13	120.6 (10)
O5—Zn2—O2^ii^	109.7 (3)	C15—C14—H14	119.7
O10—Zn2—O2^ii^	102.5 (3)	C13—C14—H14	119.7
O3—P1—O2	112.4 (4)	C14—C15—N5	120.3 (10)
O3—P1—O1	112.2 (3)	C14—C15—H15	119.9
O2—P1—O1	113.1 (4)	N5—C15—H15	119.9
O3—P1—H1P	106.1	C11—N5—C15	121.3 (8)
O2—P1—H1P	106.1	C11—N5—H7N	119.3
O1—P1—H1P	106.1	C15—N5—H7N	119.3
O6—P2—O4	113.8 (4)	C11—N6—H8N	119.9
O6—P2—O5	113.0 (4)	C11—N6—H9N	123.8
O4—P2—O5	111.0 (4)	H8N—N6—H9N	116.0
O6—P2—H2P	106.1	C12—O15—H3O	105.8
O4—P2—H2P	106.1	N8—C16—N7	119.2 (8)
O5—P2—H2P	106.1	N8—C16—C17	123.2 (8)
O9—P3—O8	111.7 (4)	N7—C16—C17	117.6 (8)
O9—P3—O7	112.0 (4)	O16—C17—C18	125.7 (8)
O8—P3—O7	112.5 (4)	O16—C17—C16	114.6 (7)
O9—P3—H3P	106.7	C18—C17—C16	119.8 (9)
O8—P3—H3P	106.7	C17—C18—C19	120.0 (9)
O7—P3—H3P	106.7	C17—C18—H18	120.0
O10—P4—O12	113.2 (4)	C19—C18—H18	120.0
O10—P4—O11	112.8 (4)	C20—C19—C18	119.7 (9)
O12—P4—O11	112.4 (4)	C20—C19—H19	120.1
O10—P4—H4P	105.9	C18—C19—H19	120.1
O12—P4—H4P	105.9	C19—C20—N7	120.3 (10)
O11—P4—H4P	105.9	C19—C20—H20	119.8
P13^i^—O1—Zn12	138.0 (7)	N7—C20—H20	119.8
P1—O1—Zn1	137.6 (4)	C16—N7—C20	122.5 (8)
P1—O2—Zn2^i^	117.7 (4)	C16—N7—H10N	118.7
P11—O4—Zn12	134.2 (6)	C20—N7—H10N	118.7
P2—O4—Zn1	139.0 (4)	C16—N8—H11N	116.9
P2—O5—Zn2	123.5 (4)	C16—N8—H12N	123.5
P3—O7—Zn2	137.6 (4)	H11N—N8—H12N	119.3
P14—O7—Zn11	133.3 (7)	C17—O16—H4O	111.9
P3—O8—Zn1^ii^	118.4 (4)	O10—Zn11—O7	136.7 (5)
Zn11—O10—P12	129.3 (6)	O10—Zn11—O28	111.9 (9)
P4—O10—Zn2	140.5 (4)	O7—Zn11—O28	101.5 (9)
P4—O11—Zn1	122.8 (4)	O10—Zn11—O24	93.0 (9)
N1—C1—N2	122.2 (8)	O7—Zn11—O24	100.1 (9)
N1—C1—C2	117.9 (8)	O28—Zn11—O24	110.8 (12)
N2—C1—C2	119.9 (8)	O4—Zn12—O1	129.8 (5)
O13—C2—C3	126.6 (8)	O4—Zn12—O25^i^	100.1 (10)
O13—C2—C1	115.0 (8)	O1—Zn12—O25^i^	103.1 (9)
C3—C2—C1	118.3 (9)	O4—Zn12—O26	110.7 (10)
C2—C3—C4	120.8 (9)	O1—Zn12—O26	101.3 (10)
C2—C3—H3	119.6	O25^i^—Zn12—O26	111.2 (13)
C4—C3—H3	119.6	O28—P11—O22	109.6 (17)
C5—C4—C3	118.3 (9)	O28—P11—O4	114.1 (13)
C5—C4—H4	120.9	O22—P11—O4	112.0 (13)
C3—C4—H4	120.9	O28—P11—H11P	106.9
N1—C5—C4	119.6 (10)	O22—P11—H11P	106.9
N1—C5—H5	120.2	O4—P11—H11P	106.9
C4—C5—H5	120.2	O21—P12—O26	112.1 (17)
C1—N1—C5	124.9 (8)	O21—P12—O10	110.4 (11)
C1—N1—H1N	117.5	O26—P12—O10	115.9 (14)
C5—N1—H1N	117.5	O21—P12—H12P	105.9
C1—N2—H2N	119.2	O26—P12—H12P	105.9
C1—N2—H3N	122.2	O10—P12—H12P	105.9
H2N—N2—H3N	117.7	O24—P13—O27	112.8 (19)
C2—O13—H1O	104.7	O24—P13—O1^ii^	110.3 (14)
N3—C6—N4	122.1 (8)	O27—P13—O1^ii^	117.8 (13)
N3—C6—C7	117.6 (8)	O24—P13—H13P	104.9
N4—C6—C7	120.3 (8)	O27—P13—H13P	104.9
O14—C7—C8	125.4 (8)	O1^ii^—P13—H13P	104.9
O14—C7—C6	114.9 (8)	O23—P14—O25	113.0 (18)
C8—C7—C6	119.7 (9)	O23—P14—O7	121.7 (13)
C7—C8—C9	120.4 (9)	O25—P14—O7	109.3 (13)
C7—C8—H8	119.8	O23—P14—H14P	103.5
C9—C8—H8	119.8	O25—P14—H14P	103.5
C10—C9—C8	118.2 (9)	O7—P14—H14P	103.5
C10—C9—H9	120.9	P13—O24—Zn11	125.8 (18)
C8—C9—H9	120.9	P14—O25—Zn12^ii^	122.0 (18)
N3—C10—C9	119.7 (10)	P12—O26—Zn12	119.5 (19)
N3—C10—H10	120.1	P11—O28—Zn11	117.6 (16)
C9—C10—H10	120.1	H1W—O1W—H2W	120.3
C6—N3—C10	124.5 (8)	H3W—O2W—H4W	116.8
C6—N3—H4N	117.8	H5W—O3W—H6W	104.0
C10—N3—H4N	117.8	H7W—O4W—H8W	130.4
C6—N4—H5N	119.3	H11W—O11W—H12W	101.5
C6—N4—H6N	112.9	H13W—O12W—H14W	102.7
H5N—N4—H6N	127.3	H15W—O13W—H16W	110.6
C7—O14—H2O	121.0	H17W—O14W—H18W	110.6
			
O3—P1—O1—Zn1	−90.4 (6)	O15—C12—C13—C14	180.0 (8)
O2—P1—O1—Zn1	38.1 (6)	C11—C12—C13—C14	−1.9 (12)
O3—P1—O2—Zn2^i^	177.4 (4)	C12—C13—C14—C15	0.1 (13)
O1—P1—O2—Zn2^i^	49.0 (5)	C13—C14—C15—N5	2.4 (13)
O6—P2—O4—Zn1	−99.7 (6)	N6—C11—N5—C15	−177.1 (7)
O5—P2—O4—Zn1	29.0 (7)	C12—C11—N5—C15	1.6 (11)
O6—P2—O5—Zn2	−168.7 (5)	C14—C15—N5—C11	−3.3 (12)
O4—P2—O5—Zn2	62.1 (6)	N8—C16—C17—O16	−1.6 (11)
O9—P3—O7—Zn2	92.4 (6)	N7—C16—C17—O16	177.8 (6)
O8—P3—O7—Zn2	−34.5 (7)	N8—C16—C17—C18	178.8 (7)
O9—P3—O8—Zn1^ii^	−178.8 (4)	N7—C16—C17—C18	−1.8 (11)
O7—P3—O8—Zn1^ii^	−51.8 (5)	O16—C17—C18—C19	−179.6 (8)
O12—P4—O10—Zn2	107.1 (6)	C16—C17—C18—C19	0.0 (12)
O11—P4—O10—Zn2	−21.9 (7)	C17—C18—C19—C20	0.3 (13)
O10—P4—O11—Zn1	−62.7 (6)	C18—C19—C20—N7	1.3 (13)
O12—P4—O11—Zn1	167.8 (4)	N8—C16—N7—C20	−177.1 (7)
N1—C1—C2—O13	−179.1 (6)	C17—C16—N7—C20	3.4 (11)
N2—C1—C2—O13	0.8 (11)	C19—C20—N7—C16	−3.3 (12)
N1—C1—C2—C3	3.0 (11)	P12—O10—Zn11—O7	42.2 (9)
N2—C1—C2—C3	−177.1 (7)	P12—O10—Zn11—O28	−95.7 (10)
O13—C2—C3—C4	−178.6 (8)	P12—O10—Zn11—O24	150.4 (10)
C1—C2—C3—C4	−1.0 (12)	P11—O4—Zn12—O1	−30.3 (9)
C2—C3—C4—C5	−2.1 (13)	P11—O4—Zn12—O25^i^	−146.9 (11)
C3—C4—C5—N1	3.2 (13)	P11—O4—Zn12—O26	95.7 (12)
N2—C1—N1—C5	178.1 (7)	P13^i^—O1—Zn12—O4	−20.5 (11)
C2—C1—N1—C5	−2.0 (12)	P13^i^—O1—Zn12—O25^i^	94.9 (12)
C4—C5—N1—C1	−1.2 (12)	P13^i^—O1—Zn12—O26	−149.9 (12)
N3—C6—C7—O14	180.0 (6)	Zn12—O4—P11—O28	−34.9 (15)
N4—C6—C7—O14	−0.1 (11)	Zn12—O4—P11—O22	90.5 (16)
N3—C6—C7—C8	1.9 (12)	Zn11—O10—P12—O21	−99.3 (13)
N4—C6—C7—C8	−178.2 (7)	Zn11—O10—P12—O26	29.5 (16)
O14—C7—C8—C9	−178.9 (8)	Zn11—O7—P14—O23	−89.2 (17)
C6—C7—C8—C9	−1.0 (12)	Zn11—O7—P14—O25	45.3 (15)
C7—C8—C9—C10	−0.7 (13)	O27—P13—O24—Zn11	168.1 (19)
C8—C9—C10—N3	1.6 (13)	O1^ii^—P13—O24—Zn11	−58 (2)
N4—C6—N3—C10	179.0 (7)	O23—P14—O25—Zn12^ii^	−165.9 (18)
C7—C6—N3—C10	−1.1 (12)	O7—P14—O25—Zn12^ii^	55 (2)
C9—C10—N3—C6	−0.7 (13)	O21—P12—O26—Zn12	179.3 (17)
N6—C11—C12—O15	−1.9 (11)	O10—P12—O26—Zn12	51 (2)
N5—C11—C12—O15	179.4 (6)	O22—P11—O28—Zn11	−175.2 (17)
N6—C11—C12—C13	179.6 (7)	O4—P11—O28—Zn11	−48.6 (18)
N5—C11—C12—C13	1.0 (11)		

Symmetry codes: (i) *x*, *y*−1, *z*; (ii) *x*, *y*+1, *z*.

### catena-Poly[bis(2-amino-3-hydroxypyridinium) [zinc-di-µ-phosphonato] dihydrate] (I) Hydrogen-bond geometry (Å, º)

**Table:**

*D*—H···*A*	*D*—H	H···*A*	*D*···*A*	*D*—H···*A*
C5—H5···O1*W*^iii^	0.95	2.60	3.539 (13)	169
N1—H1*N*···O9	0.88	1.73	2.595 (10)	168
N2—H2*N*···O16^iv^	0.85	2.31	3.060 (10)	147
N2—H3*N*···O8	0.87	2.10	2.926 (9)	157
O13—H1*O*···O6^iv^	0.96	1.58	2.488 (9)	156
C8—H8···O3*W*^iv^	0.95	2.55	3.225 (13)	128
C9—H9···O4*W*^v^	0.95	2.61	3.562 (12)	178
C10—H10···O3^iii^	0.95	2.54	3.486 (14)	171
N3—H4*N*···O2*W*	0.88	1.93	2.771 (10)	160
N4—H5*N*···O15^iv^	0.86	2.22	2.959 (10)	144
N4—H6*N*···O11	0.94	2.01	2.853 (9)	148
O14—H2*O*···O3*W*^iv^	0.94	1.78	2.656 (9)	154
C14—H14···O3*W*^vi^	0.95	2.54	3.490 (12)	175
C15—H15···O9^vi^	0.95	2.50	3.442 (14)	172
N5—H7*N*···O1*W*	0.88	1.91	2.756 (9)	162
N6—H8*N*···O13^vii^	0.91	2.19	3.019 (9)	151
N6—H9*N*···O5	0.91	1.97	2.824 (10)	156
O15—H3*O*···O12^vii^	0.95	1.57	2.474 (9)	157
C18—H18···O4*W*^vii^	0.95	2.47	3.134 (12)	127
C20—H20···O2*W*^vi^	0.95	2.59	3.508 (13)	163
N7—H10*N*···O3	0.88	1.73	2.595 (10)	169
N8—H11*N*···O14^viii^	0.91	2.30	3.090 (9)	145
N8—H12*N*···O2	0.85	2.21	3.012 (9)	157
O16—H4*O*···O4*W*^vii^	0.92	1.72	2.611 (10)	161
O1*W*—H1*W*···O10	0.72	2.13	2.787 (9)	152
O1*W*—H2*W*···O3	0.80	1.94	2.732 (8)	171
O2*W*—H3*W*···O4	0.84	2.04	2.785 (9)	147
O2*W*—H4*W*···O9	0.89	1.83	2.711 (9)	168
O3*W*—H5*W*···O6^ii^	0.91	1.79	2.694 (9)	177
O3*W*—H6*W*···O7	0.84	1.96	2.797 (9)	180
O4*W*—H7*W*···O12	0.93	1.77	2.694 (9)	172
O4*W*—H8*W*···O1^ii^	0.80	2.03	2.760 (9)	150
O11*W*—H11*W*···O22^ii^	0.82	1.90	2.72 (4)	178
O11*W*—H12*W*···O7	0.78	1.87	2.66 (4)	178
O12*W*—H13*W*···O1	0.80	1.84	2.65 (3)	179
O12*W*—H14*W*···O21^i^	0.83	1.98	2.81 (3)	179
O13*W*—H15*W*···O4	0.80	1.88	2.68 (3)	178
O13*W*—H16*W*···O23	0.83	1.91	2.74 (4)	177
O14*W*—H17*W*···O10	0.82	1.92	2.73 (4)	176
O14*W*—H18*W*···O27^i^	0.80	1.92	2.72 (4)	176

Symmetry codes: (i) *x*, *y*−1, *z*; (ii) *x*, *y*+1, *z*; (iii) *x*−1, *y*, *z*; (iv) *x*−1/2, −*y*+1, *z*−1/2; (v) *x*−1, *y*−1, *z*; (vi) *x*+1, *y*, *z*; (vii) *x*+1/2, −*y*+1, *z*+1/2; (viii) *x*+1/2, −*y*, *z*+1/2.

### Poly[(benzene-1,2-diamine)(µ5-phosphonato)zinc] (II) Crystal data

**Table:**

[Zn(HPO_3_)(C_6_H_8_N_2_)]	*D*_x_ = 1.907 Mg m^−^^3^
*M**_r_* = 253.49	Mo *K*α radiation, λ = 0.71073 Å
Orthorhombic, *P**c**a*2_1_	Cell parameters from 8070 reflections
*a* = 8.0419 (2) Å	θ = 2.9–28.8°
*b* = 13.5008 (4) Å	µ = 2.94 mm^−^^1^
*c* = 8.1307 (2) Å	*T* = 173 K
*V* = 882.77 (4) Å^3^	Plate, colourless
*Z* = 4	0.27 × 0.10 × 0.02 mm
*F*(000) = 512	

### Poly[(benzene-1,2-diamine)(µ5-phosphonato)zinc] (II) Data collection

**Table:**

Rigaku XtaLAB P200 HPC diffractometer	2038 independent reflections
Radiation source: rotating_anode, Rigaku FR-X	1952 reflections with *I* > 2σ(*I*)
Rigaku Osmic Confocal Optical System monochromator	*R*_int_ = 0.044
ω scans	θ_max_ = 29.2°, θ_min_ = 3.0°
Absorption correction: multi-scan (CrysAlis PRO; Rigaku OD, 2017)	*h* = −10→10
*T*_min_ = 0.731, *T*_max_ = 1.000	*k* = −17→16
11004 measured reflections	*l* = −10→10

### Poly[(benzene-1,2-diamine)(µ5-phosphonato)zinc] (II) Refinement

**Table:**

Refinement on *F*^2^	Hydrogen site location: mixed
Least-squares matrix: full	H atoms treated by a mixture of independent and constrained refinement
*R*[*F*^2^ > 2σ(*F*^2^)] = 0.022	*w* = 1/[σ^2^(*F*_o_^2^) + (0.0292*P*)^2^] where *P* = (*F*_o_^2^ + 2*F*_c_^2^)/3
*wR*(*F*^2^) = 0.051	(Δ/σ)_max_ < 0.001
*S* = 1.03	Δρ_max_ = 0.71 e Å^−^^3^
2038 reflections	Δρ_min_ = −0.27 e Å^−^^3^
131 parameters	Absolute structure: Flack (1983) parameter
1 restraint	Absolute structure parameter: 0.016 (14)
Primary atom site location: structure-invariant direct methods	

### Poly[(benzene-1,2-diamine)(µ5-phosphonato)zinc] (II) Special details

**Table:**

Geometry. All esds (except the esd in the dihedral angle between two l.s. planes) are estimated using the full covariance matrix. The cell esds are taken into account individually in the estimation of esds in distances, angles and torsion angles; correlations between esds in cell parameters are only used when they are defined by crystal symmetry. An approximate (isotropic) treatment of cell esds is used for estimating esds involving l.s. planes.
Refinement. Refined as a 2-component inversion twin

### Poly[(benzene-1,2-diamine)(µ5-phosphonato)zinc] (II) Fractional atomic coordinates and isotropic or equivalent isotropic displacement parameters (Å^2^)

**Table:**

	*x*	*y*	*z*	*U*_iso_*/*U*_eq_	
Zn1	0.34696 (3)	0.06830 (2)	0.34354 (5)	0.01300 (11)	
P1	0.00036 (8)	0.12113 (5)	0.21567 (10)	0.01250 (16)	
H1	−0.0400	0.2127	0.1753	0.015*	
O1	0.1265 (2)	0.12693 (13)	0.3561 (3)	0.0175 (4)	
O2	−0.1583 (2)	0.06927 (12)	0.2738 (3)	0.0164 (5)	
O3	0.0745 (3)	0.07310 (14)	0.0661 (3)	0.0229 (5)	
C1	0.5193 (3)	0.2527 (2)	0.2485 (4)	0.0149 (6)	
C2	0.4078 (3)	0.3236 (2)	0.1875 (4)	0.0181 (6)	
C3	0.4181 (4)	0.4194 (2)	0.2496 (4)	0.0235 (7)	
H3	0.3426	0.4681	0.2106	0.028*	
C4	0.5345 (4)	0.4458 (2)	0.3662 (5)	0.0269 (7)	
H4	0.5386	0.5119	0.4059	0.032*	
C5	0.6460 (4)	0.3756 (3)	0.4257 (4)	0.0263 (8)	
H5	0.7264	0.3931	0.5062	0.032*	
C6	0.6374 (3)	0.2797 (2)	0.3651 (5)	0.0202 (7)	
H6	0.7138	0.2314	0.4041	0.024*	
N1	0.5069 (3)	0.14987 (17)	0.2000 (4)	0.0155 (5)	
H1N	0.601 (4)	0.121 (2)	0.199 (5)	0.019*	
H2N	0.465 (4)	0.139 (2)	0.113 (4)	0.019*	
N2	0.2847 (3)	0.2972 (2)	0.0758 (4)	0.0261 (7)	
H3N	0.230 (5)	0.343 (3)	0.039 (5)	0.031*	
H4N	0.311 (4)	0.250 (3)	0.025 (5)	0.031*	

### Poly[(benzene-1,2-diamine)(µ5-phosphonato)zinc] (II) Atomic displacement parameters (Å^2^)

**Table:**

	*U*^11^	*U*^22^	*U*^33^	*U*^12^	*U*^13^	*U*^23^
Zn1	0.01069 (16)	0.01757 (17)	0.01076 (17)	−0.00001 (9)	−0.00039 (16)	−0.00114 (16)
P1	0.0106 (3)	0.0163 (3)	0.0105 (4)	0.0005 (2)	0.0004 (3)	0.0012 (3)
O1	0.0137 (8)	0.0240 (9)	0.0148 (11)	0.0020 (6)	−0.0017 (10)	−0.0028 (11)
O2	0.0092 (11)	0.0186 (12)	0.0214 (13)	0.0005 (6)	0.0007 (7)	0.0033 (8)
O3	0.0256 (13)	0.0302 (13)	0.0130 (12)	0.0011 (8)	0.0044 (9)	−0.0023 (8)
C1	0.0144 (13)	0.0176 (13)	0.0127 (14)	−0.0031 (10)	0.0054 (11)	0.0018 (10)
C2	0.0158 (13)	0.0225 (14)	0.0160 (15)	−0.0001 (11)	0.0036 (12)	0.0032 (11)
C3	0.0272 (18)	0.0223 (14)	0.0210 (17)	0.0020 (12)	0.0048 (14)	0.0039 (11)
C4	0.0350 (15)	0.0215 (14)	0.024 (2)	−0.0052 (12)	0.0038 (16)	−0.0063 (14)
C5	0.0244 (16)	0.0315 (19)	0.0230 (19)	−0.0061 (13)	−0.0028 (12)	−0.0056 (14)
C6	0.0168 (13)	0.0249 (14)	0.019 (2)	0.0000 (10)	0.0000 (12)	0.0007 (14)
N1	0.0140 (11)	0.0197 (11)	0.0128 (14)	0.0028 (9)	−0.0015 (10)	0.0001 (11)
N2	0.0288 (19)	0.0244 (13)	0.0250 (16)	0.0087 (11)	−0.0079 (14)	−0.0021 (12)

### Poly[(benzene-1,2-diamine)(µ5-phosphonato)zinc] (II) Geometric parameters (Å, º)

**Table:**

Zn1—O3^i^	1.918 (2)	C2—N2	1.389 (4)
Zn1—O2^ii^	1.9425 (17)	C2—C3	1.390 (4)
Zn1—O1	1.9445 (16)	C3—C4	1.379 (5)
Zn1—N1	2.056 (3)	C3—H3	0.9500
P1—O3	1.501 (2)	C4—C5	1.391 (5)
P1—O1	1.529 (3)	C4—H4	0.9500
P1—O2	1.5299 (19)	C5—C6	1.388 (5)
P1—H1	1.3200	C5—H5	0.9500
O2—Zn1^iii^	1.9424 (17)	C6—H6	0.9500
O3—Zn1^iv^	1.918 (2)	N1—H1N	0.85 (3)
C1—C6	1.390 (4)	N1—H2N	0.80 (3)
C1—C2	1.402 (4)	N2—H3N	0.82 (4)
C1—N1	1.447 (4)	N2—H4N	0.79 (4)
			
O3^i^—Zn1—O2^ii^	108.36 (9)	C4—C3—C2	122.0 (3)
O3^i^—Zn1—O1	103.71 (10)	C4—C3—H3	119.0
O2^ii^—Zn1—O1	112.63 (7)	C2—C3—H3	119.0
O3^i^—Zn1—N1	108.15 (11)	C3—C4—C5	120.1 (3)
O2^ii^—Zn1—N1	111.10 (10)	C3—C4—H4	120.0
O1—Zn1—N1	112.46 (9)	C5—C4—H4	120.0
O3—P1—O1	111.30 (12)	C6—C5—C4	118.7 (3)
O3—P1—O2	112.56 (12)	C6—C5—H5	120.7
O1—P1—O2	110.24 (13)	C4—C5—H5	120.7
O3—P1—H1	107.5	C5—C6—C1	121.3 (3)
O1—P1—H1	107.5	C5—C6—H6	119.3
O2—P1—H1	107.5	C1—C6—H6	119.3
P1—O1—Zn1	123.02 (15)	C1—N1—Zn1	113.73 (19)
P1—O2—Zn1^iii^	120.64 (10)	C1—N1—H1N	112 (2)
P1—O3—Zn1^iv^	155.43 (13)	Zn1—N1—H1N	109 (2)
C6—C1—C2	120.0 (3)	C1—N1—H2N	117 (2)
C6—C1—N1	118.9 (2)	Zn1—N1—H2N	98 (2)
C2—C1—N1	121.0 (2)	H1N—N1—H2N	106 (3)
N2—C2—C3	121.2 (3)	C2—N2—H3N	115 (2)
N2—C2—C1	120.8 (3)	C2—N2—H4N	111 (2)
C3—C2—C1	117.9 (3)	H3N—N2—H4N	124 (3)
			
O3—P1—O1—Zn1	−4.93 (18)	N2—C2—C3—C4	177.0 (3)
O2—P1—O1—Zn1	120.71 (14)	C1—C2—C3—C4	0.8 (5)
O3—P1—O2—Zn1^iii^	61.4 (2)	C2—C3—C4—C5	−0.2 (5)
O1—P1—O2—Zn1^iii^	−63.54 (18)	C3—C4—C5—C6	0.2 (5)
O1—P1—O3—Zn1^iv^	−114.0 (4)	C4—C5—C6—C1	−0.7 (5)
O2—P1—O3—Zn1^iv^	121.6 (3)	C2—C1—C6—C5	1.2 (5)
C6—C1—C2—N2	−177.5 (3)	N1—C1—C6—C5	−174.8 (3)
N1—C1—C2—N2	−1.5 (4)	C6—C1—N1—Zn1	89.0 (3)
C6—C1—C2—C3	−1.2 (4)	C2—C1—N1—Zn1	−87.0 (3)
N1—C1—C2—C3	174.7 (3)		

Symmetry codes: (i) −*x*+1/2, *y*, *z*+1/2; (ii) *x*+1/2, −*y*, *z*; (iii) *x*−1/2, −*y*, *z*; (iv) −*x*+1/2, *y*, *z*−1/2.

### Poly[(benzene-1,2-diamine)(µ5-phosphonato)zinc] (II) Hydrogen-bond geometry (Å, º)

**Table:**

*D*—H···*A*	*D*—H	H···*A*	*D*···*A*	*D*—H···*A*
N1—H1*N*···O2^v^	0.85 (3)	2.15 (4)	2.966 (3)	161 (3)
N1—H2*N*···O1^iv^	0.80 (3)	2.22 (4)	3.011 (4)	171 (3)
N2—H4*N*···O1^iv^	0.79 (4)	2.21 (4)	2.998 (4)	174 (4)
N2—H3*N*···*Cg*^iv^	0.82 (4)	2.80 (4)	3.400 (3)	132 (3)

Symmetry codes: (iv) −*x*+1/2, *y*, *z*−1/2; (v) *x*+1, *y*, *z*.
